# Epifaunal Assemblages of the Fan Mussel *Atrina fragilis* (Mollusca: Bivalvia) in the Sea of Marmara

**DOI:** 10.3390/biology14080945

**Published:** 2025-07-27

**Authors:** Melih Ertan Çinar, Mehmet Baki Yokeş, Deniz Erdogan-Dereli, Sermin Açik, Alper Evcen

**Affiliations:** 1 Department of Hydrobiology, Faculty of Fisheries, Ege University, Bornova, Izmir 35100, Türkiye; dnzer09@gmail.com; 2 SERPULA Marine Research Ltd., Co., Gülbahce Mahallesi, Gülbahce Caddesi, Urla, Izmir 35100, Türkiye; 3 AMBRD Doğa Bilimleri, Hanımefendi Sokak 160/6, Şişli, İstanbul 34384, Türkiye; bakiyokes@gmail.com; 4 Institute of Marine Sciences and Technology, Dokuz Eylül University, Balçova 35330, Türkiye; sermin.acikcinar@deu.edu.tr; 5 The Scientific and Technological Research Council of Türkiye (TÜBİTAK) Marmara Research Center, Gebze, Kocaeli 41470, Türkiye; alper.evcen@tubitak.gov.tr

**Keywords:** *Atrina fragilis*, Pinnidae, population density, epifaunal associations, biogenic habitat, Sea of Marmara

## Abstract

Dense aggregations of species in the family Pinnidae give soft substrata a specific characterization. They may influence the biological and physical properties of the surrounding sediments. Bottom-trawl samplings performed in the Sea of Marmara revealed populations of a large pinnid species, particularly at depths of 40–45 m in soft substrata. Both morphological and DNA analyses confirmed the species’ taxonomic identity as *Atrina fragilis*. This species had a population density ranging from 31 to 469 ind.km^−2^, and the shell lengths ranged from 21.3 to 31 cm. A total of 47 macrozoobenthic species belonging to eight taxonomic groups were found on the shells of ten live and nine dead *A. fragilis* individuals. Polychaeta accounted for 53% of the total number of species and 75% of the total number of individuals. Among these species, *Protula tubularia* and *Serpula concharum* comprised almost 30% of all epifaunal populations. Community parameters changed according to the shell length and width. Different faunal assemblages were encountered on the shells. Given the ecological significance of *A. fragilis* as both a habitat-forming and sensitive benthic species, conservation measures should prioritize the protection of known habitats and the regulation of activities that lead to seabed disturbance.

## Simple Summary

The fan mussel *Atrina fragilis* forms dense beds at certain depths and substrata, creating reef-like projections on soft substrata that attract a variety of species. The distribution of this species and its associated fauna has been studied little in the Mediterranean Sea. This paper presents the biometric features and population density of the species around the southern Marmara Islands in the Sea of Marmara. Examining the macrofaunal components on the shells of dead and live *A. fragilis* individuals revealed high species richness, with one or two serpulid species being dominant. Shell size appears to be an important factor in the community parameters. Long-term monitoring programs are essential for clarifying changes in the distribution and epifaunal components of *A. fragilis* when exposed to different stressors.

## 1. Introduction

Large bivalves inhabiting soft substrata patchily create reef-like projections that attract settlements of a variety of species and function as ecosystem engineers [1,2,3,4]. In the Mediterranean Sea, these biogenic habitats are formed by species in the Pinnidae family, such as *Pinna nobilis* (Linnaeus, 1758), *P. rudis* (Linnaeus, 1758), and *Atrina fragilis* (Pennant, 1777). The morphological features and ecological requirements of these species differ considerably. *Pinna nobilis* has a maximum shell length of 120 cm and primarily inhabits shallow-water, soft-bottom environments with seagrasses across the Mediterranean Sea, including the Sea of Marmara, at depths of up to 60 m [5,6]. *Pinna rudis* has a maximum shell length of 50 cm and primarily occurs in gravel bottoms and hard substrata, frequently appearing in the western Mediterranean Sea and the eastern Atlantic Ocean at depths of up to 40 m, but it tends to expand its distributional range to the eastern Mediterranean [7,8]. *Atrina fragilis* has a maximum shell length of 50 cm and preferably inhabits mud and sandy mud bottoms at depths of up to 600 m in the eastern Atlantic Ocean and Mediterranean Sea [9,10]. Pérès and Picard [11] indicated that *A. fragilis* mainly occurs in the zone between 30 and 200 m in the regions. However, the depth distribution of this species can vary depending on the turbidity; more turbid water enables this species to inhabit shallower depths [12]. It occurs primarily in the detritic or muddy sand habitats [9,13]. *Atrina fragilis* has been reported in all seas surrounding Türkiye, except for the Black Sea [14,15].

*Atrina fragilis* has been frequently reported at depths between 25 and 50 m in the Mediterranean Sea. Like other pinnid bivalves, it has distinctive fan-shaped shells that are firmly anchored in soft substrata by byssus threads, with typically one-third of the shell embedded in the sediment [13]. In the Aegean Sea, the growth rate and lifespan of *A. fragilis* were estimated as 1.2–2.6 cm per year and 32 years, respectively [16]. It is not targeted for consumption but is caught as bycatch in fishing activities. Seabed-skimming fishing gear such as dredges and trawls can have an adverse effect on its populations and cause them to disappear in areas with heavy fishing activity. For example, trawling has led to a 70% decrease in the *A. fragilis* population in the Gulf of Venice [17]. Therefore, some national legal measures have been taken in the eastern Atlantic to protect it [18]. Although the EUNIS page (https://eunis.eea.europa.eu/references/1566/species, accessed on 15 May 2025) states that this species is classified as a strictly protected fauna species under the Bern Convention (Annex II, Amendment), the Bern Convention list actually refers to *Pinna pernula*, a synonym of *Pinna rudis* rather than *A. fragilis* [19].

The high occurrence of pinnid species gives soft substrata a specific characterization. The feeding activity of these large bivalves can influence the biological and physical properties of the surrounding sediments [20]. Their physical structure can modify water flow in soft substrata [21]. Soft-bottom species assemblages inside and outside the *Atrina* bed were found to be different, indicating its role in structuring the biotic assemblages in the habitat [20,22]. The shells of these species are home to diverse fauna belonging to different groups, from sponges to fish. Associated species use the shells as a substratum, shelter, and nesting site [23,24].

Several attempts have been carried out to elucidate the species diversity associated with live and dead *P. nobilis* [1,3,24,25,26,27] and *P. rudis* [28] shells, but the epifaunal components of *A. fragilis* have not yet been studied in detail. This study aims to elucidate the distribution and population characteristics of *A. fragilis* around the southern Marmara Islands in the Sea of Marmara, and to determine the diversity of its associated macrobenthic epifauna.

## 2. Materials and Methods

### 2.1. Sampling of Epifauna

**Figure 1:**
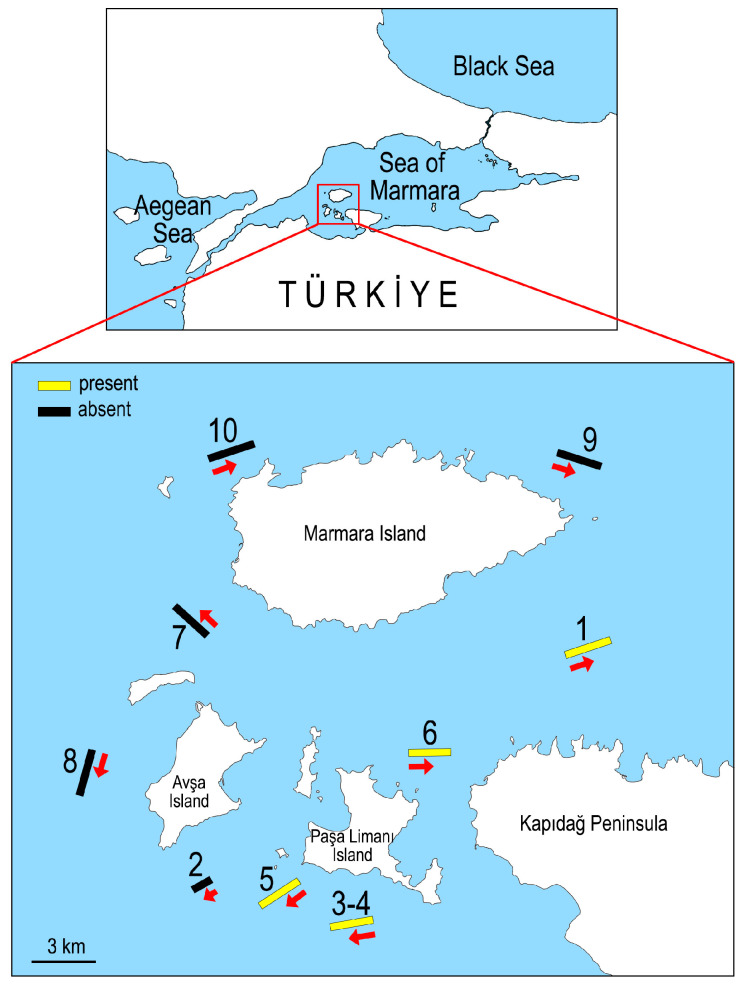
Map of the investigated area with the locations of the bottom trawl stations. The stations where *Atrina fragilis* were found are indicated in yellow. The arrow shows the direction of the bottom trawling hauls.

As part of the MARIAS project (Addressing Invasive Alien Species Threats at Key Marine Biodiversity Areas Project), bottom trawling was conducted at ten stations around the southern Marmara Islands to determine the depth preferences of the invasive alien species in different seasons (spring, fall, and winter) of 2021 and 2022 (see [29]). Of these stations, *Atrina fragilis* populations were only found at five stations (stations 1, 3–6) in 2021 (Table 1, Figure 1).

The bottom trawl used was a Mediterranean-type trawl, with a cod end mesh size of 20 mm and a head rope length of approximately 28 m. Each haul at each station lasted 30 min at a speed of 2.5 knots.

The following equation was used to estimate the area swept by the bottom trawl: a = V.t.hr.X2, where a is the swept area, V is the towing velocity, t is the trawl duration, hr is the head rope length, and X2 is the fraction of the head rope length (0.5 was used in this study) [30]. The swept area of each trawling haul was estimated to be 0.032 km^2^ [29].

The population density of the species was estimated by dividing its abundance by the swept area (0.032 km^2^). The biometric features of the populations were characterized by measuring the shell length (from the umbo to the posterior end) and width (the widest measurement in the posterior part) using a caliper, and weighing live individuals using a digital balance with 0.1 sensitivity.

At station 4, only individuals (3 dead and 9 live individuals) with unbroken shells were selected for the study of the epifaunal assemblages of *A. fragilis*. The epifauna associated with the shells of each dead and live specimen of *A. fragilis* at this station were carefully scraped off using a knife and spatula and placed in separate vials containing 4% formaldehyde. As the shells of *A. fragilis* at station 5 were either partly or completely broken, it was not possible to collect the epifauna separately from the shells. Therefore, the scraped material from all *A. fragilis* individuals (6 dead and 1 live individuals) at station 5 was placed in a large jar containing 4% formaldehyde. As all individuals at the other stations were completely broken, the epifaunal components of shells at these stations were not taken into account.

In the laboratory, the scraped material was washed under tap water and sorted under a stereomicroscope. The specimens belonging to different groups in the samples were identified and counted.

Based on the species-sample data matrix (including eight live and three dead individuals of *A. fragilis*), the community parameters such as the number of species (S), the number of individuals (N), the Shannon–Weaver diversity index (H′), and the Pielou’s evenness index (J′) were estimated in order to characterize the faunal communities on the shells. Faunal data on the shells of one live individual of *A. fragilis* at station 4 were omitted from the univariate and multivariate analyses, because only one colony of the ascidian *Didemnum* sp. was found on the shells. The length-weight, and the width-weight relationships were explored by using exponential regression analysis. In the regression formula (W = aX^b^), W is the wet weight of the shell in grams, X is the length or width of the shell, and a and b are coefficients related to body form. When b equals to 3, it indicates isometric growth, i.e., organs grow at the same rate as the body growth. Pearson’s product–moment correlation coefficient analysis was then employed to determine the relationships between shell length and width, and the community parameters. A polynomial regression analysis was performed to quantify the relationship between shell length and width, and the community parameters. A Student’s *t*-test was performed to determine whether there were significant differences in faunal community parameters between dead and live individuals.

The multivariate assemblage pattern was explored and visualized using Principal Coordinate Analysis (PCoA) [31], which was applied to Bray–Curtis resemblance matrices at the species level. Similarity Percentages Analysis (SIMPER) [32] was then applied to the species matrices to identify the species contributing most to the similarity between groups of samples. ANOSIM (Analysis of Similarities) was carried out to test for differences in the assemblages on the shells. All statistical analyses were performed by using Excel and PRIMER 7 [33].

### 2.2. Genetic Analysis

At station 4, tissue samples were taken from two *A. fragilis* individuals and placed in a vial containing absolute ethanol for molecular analysis. In the laboratory, DNA was extracted from the tissue fragments using a Genomic DNA Isolation Kit (AMBRD), following the instructions in the user manual. The mitochondrial cytochrome c oxidase subunit I (COI) sequence was then partially amplified from the isolated DNA using PCR. The PCR mixture included 10 ng of genomic DNA, 5 μL of 5X PCR Master Mix (AMBRD), augmented with MgCl_2_ to a final concentration of 2.0 mM and 0.1 μM of each primer, giving a final volume of 25 μL. The PCR steps were as follows: initial denaturation at 95 °C for 2 min, followed by 35 cycles of denaturation at 95 °C for 30 s, annealing at 40 °C for 1 min, and extension at 72 °C for 1 min. The PCR was completed with a final extension step at 72 °C for 5 min. The LCO1490 and HCO2198 primers were used [34]. The products were sequenced using LCO1490 (Macrogen Europe, Amsterdam, The Netherlands). The sequences have been deposited in the NCBI GenBank database under the accession numbers PV163305 and PV164315.

## 3. Results

### 3.1. Species Identification

**Figure 2:**
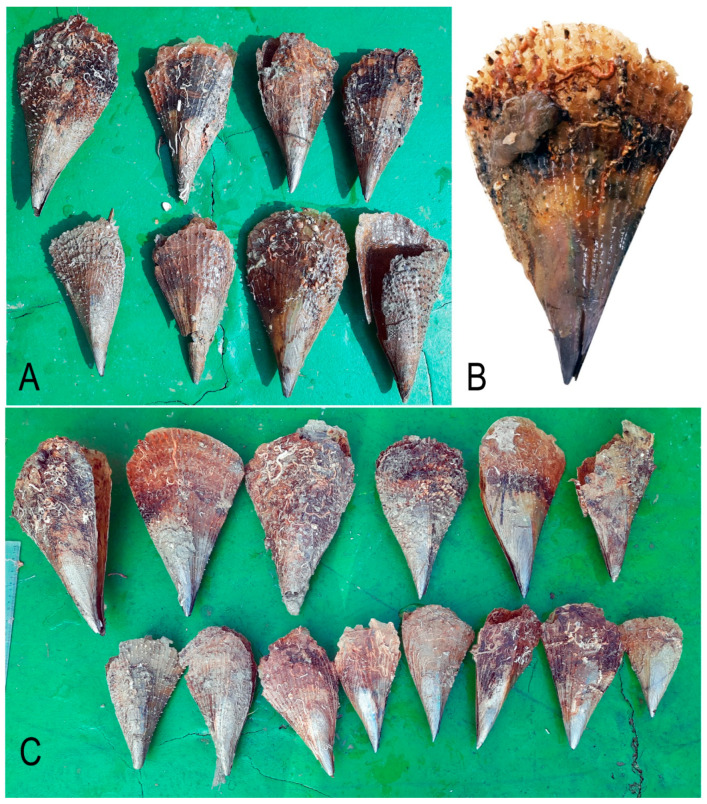
Dead and live *Atrina fragilis* individuals at stations 4 (**B**,**C**) and 5 (**A**).

Morphological and DNA analyses indicated that the large bivalve individuals inhabiting depths of 42 and 67 m around the southern Marmara Islands belonged to the pinnid species *Atrina fragilis*. The shell is somewhat triangular in shape. The outer surface of the shell is slightly convex and bears 20–55 protruding radial ribs that extend from the umbo towards the posterior margin (Figure 2). Sequencing of the COI gene resulted in the reading of 641 base pairs. A NCBI BLAST search (1.4.0) [35] revealed a 100% match with the *A. fragilis* sequences from Malaga, Spain, western Mediterranean (accession number: AF120648), and an unspecified area (accession number: KJ366406).

### 3.2. The Population Features of Atrina fragilis

**Figure 3:**
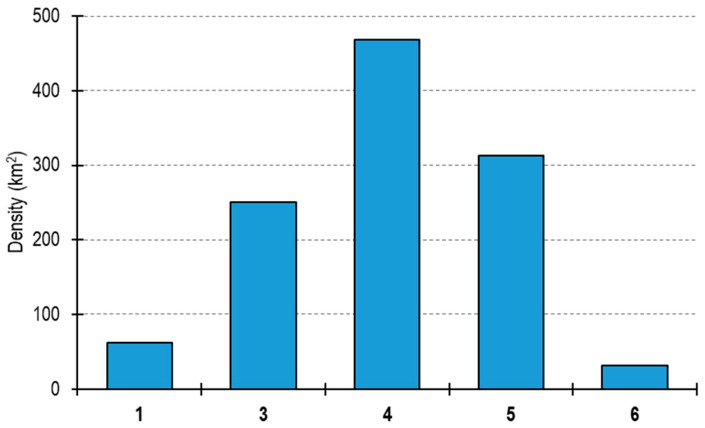
The population density of *Atrina fragilis* at stations around the southern Marmara Islands.

*Atrina fragilis* was found at five out of ten bottom trawling stations around the southern Marmara Islands. The abundance of this species varied from one individual at station 6 to 15 individuals at station 4. The estimated population density of the species ranged from 31 ind.km^−2^ at station 6 to 469 ind.km^−2^ at station 4 (Figure 3).

**Figure 4:**
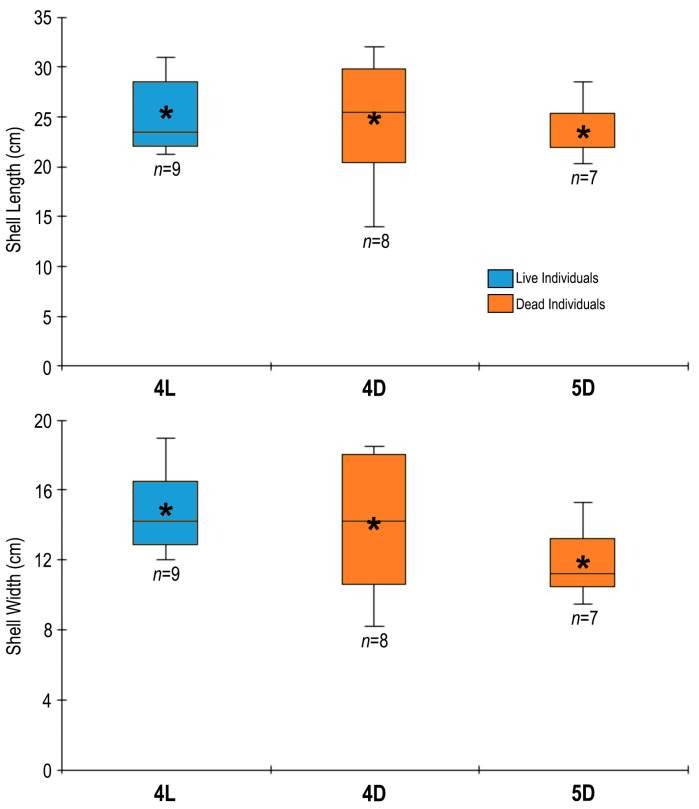
The median, mean (asterisk), quartiles (box), and minimum–maximum values of shell length (upper graph) and width (lower graph) of live (L) and dead (D) individuals of *Atrina fragilis* at stations.

The shell lengths of live individuals (*n* = 9) at station 4 ranged from 21.3 cm to 31 cm (median: 23.5 cm, mean: 25.31 cm) (Figure 4). The median value of the shell length of dead individuals (*n* = 8) at station 4 (25.5 cm) was higher than that (*n* = 7) at station 5 (22 cm). The shell width of live specimens at station 4 varied between 12 cm and 19 cm (median: 14.2 cm, mean: 14.8 cm) (Figure 4). The mean shell width of dead individuals at station 5 (11.8 cm) was lower than that at station 4 (14.05 cm). The wet weights of *A. fragilis* at station 4 ranged from 116 g to 366 g (mean: 210.6 g, median: 180 g).

**Figure 5:**
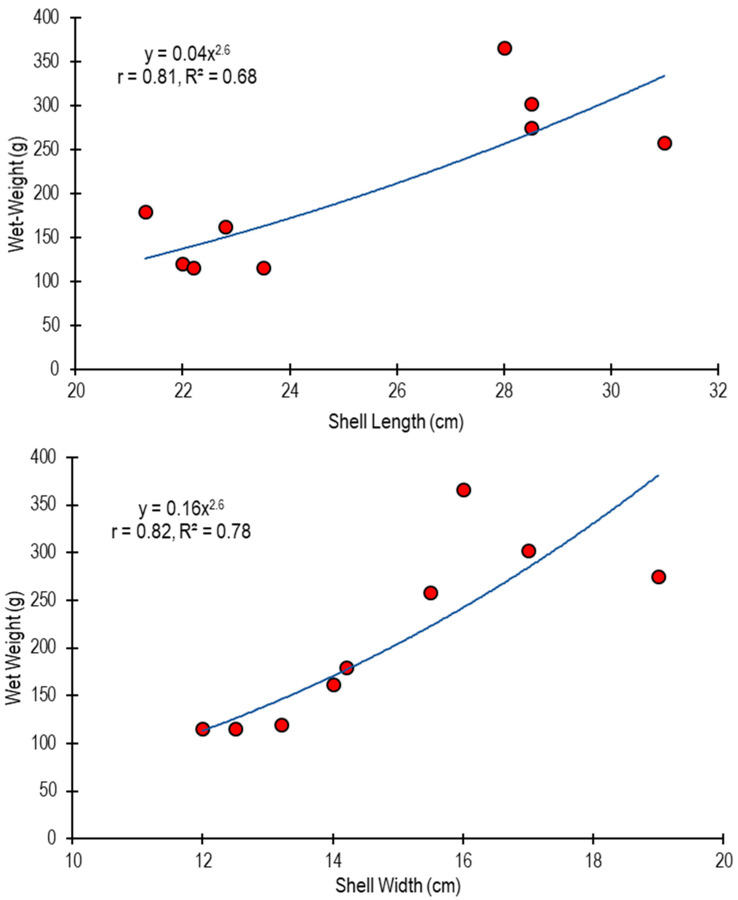
The shell length–wet weight (upper graph) and the shell width–wet weight (lower graph) relationships of the *Atrina fragilis* population in the Sea of Marmara (live individuals at station 4).

Based on live individuals (N = 9) of *Atrina fragilis* collected at station 4, strong, significant exponential relationships were estimated between shell length (r = 0.81, *p* < 0.05) and shell width (r = 0.82, *p* < 0.05), and wet weight (Figure 5). The determination coefficient of the regression equation was relatively high (R^2^ > 0.67). According to the b-value (2.47) in the length-weight relationship equation, *A. fragilis* exhibited an allometric growth.

### 3.3. Epifauna on Atrina fragilis Shell

A total of 261 invertebrate individuals/colonies belonging to 47 species and eight taxonomic groups (Porifera, Cnidaria, Polychaeta, Crustacea, Mollusca, Bryozoa, Echinodermata, and Tunicata) were found in scraped material from the shells of 10 live and nine dead *Atrina fragilis* individuals collected at stations 4 and 5 (Table 2). Based on data collected separately from *A. fragilis* individuals at station 4, only 62 individuals belonging to 17 species and six groups were found on the shells of three dead individuals, whereas 143 individuals belonging to 37 species and eight groups were obtained on the shells of eight live individuals. Some specimens could only be identified at the genus level (e.g., *Gnathia* sp., *Benedenipora* sp. and *Aphelochaeta* sp.) due to morphological differences with the known species, discrepancies with the previously described species, and the missing body parts, particularly in polychaetes. Several individuals of the foraminiferan species *Valvulineria bradyana* (Fornasini, 1900) were observed on the tubes of the serpulid polychaete *Hydroides norvegica*, but these were not included in the analysis.

**Figure 6:**
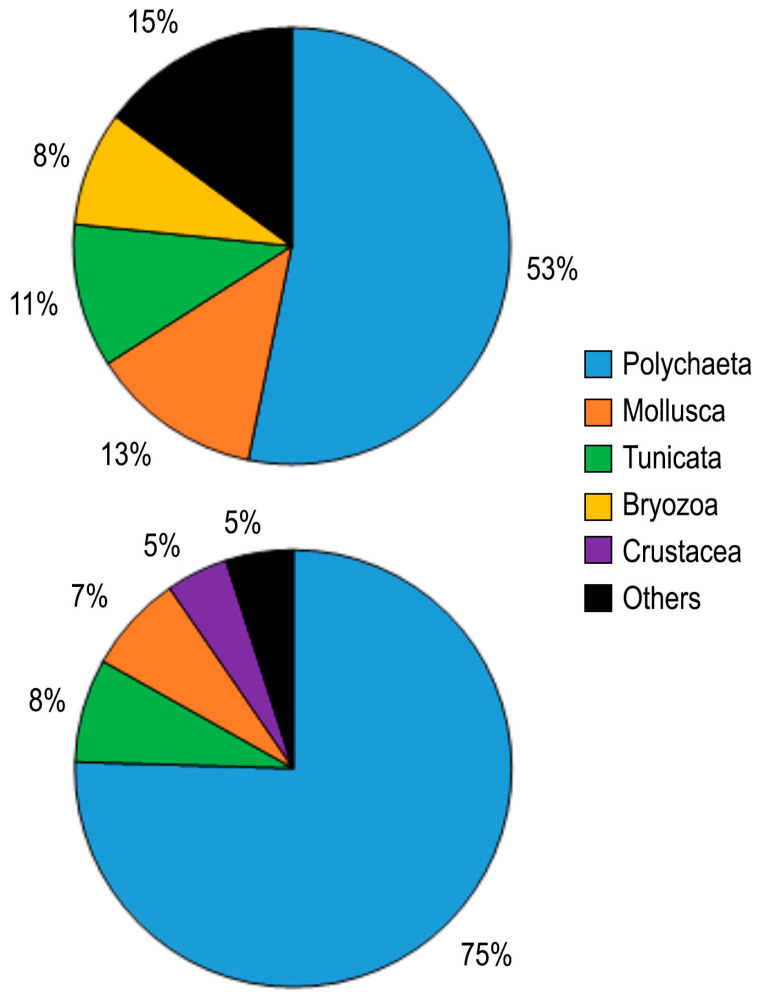
Relative dominance of taxonomic groups on shells of *Atrina fragilis* in terms of the number of species (upper graph) and the number of individuals (lower graph).

Based on data collected from all *Atrina fragilis* shells, Polychaeta ranked first in terms of both the number of species (25 species, comprising 53% of total number of species) and individuals (197 individuals, 75% of the total number of individuals), followed by Mollusca and Tunicata (Figure 6). The dominant group and its percentage remained unchanged in both dead and live shells, accounting for over 70% of individuals.

A total of 12 polychaete families were found on the shells of *Atrina fragilis*, among which Serpulidae were clearly dominant in terms of the number of individuals, accounting for 39% of the total epifaunal population and 51% of the total polychaete population. Terebellidae and Cirratulidae were represented by a relatively high number of species (four species), but the abundance of Cirratulidae (1.5% of all individuals) was considerably lower than that of Terebellidae (14%).

**Figure 7:**
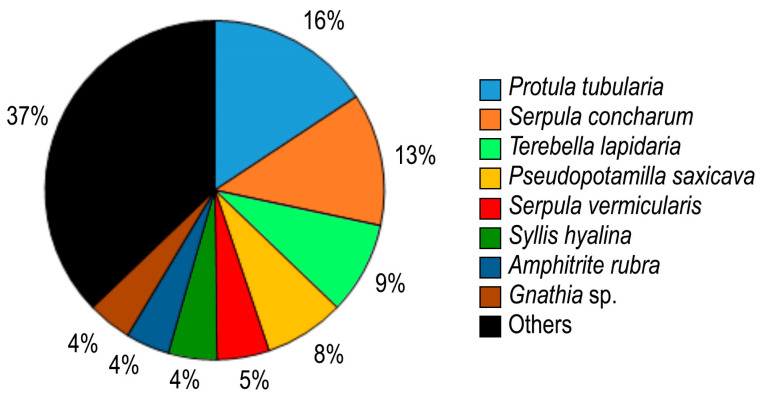
Dominant epifaunal species on shells of *Atrina fragilis*.

**Figure 8:**
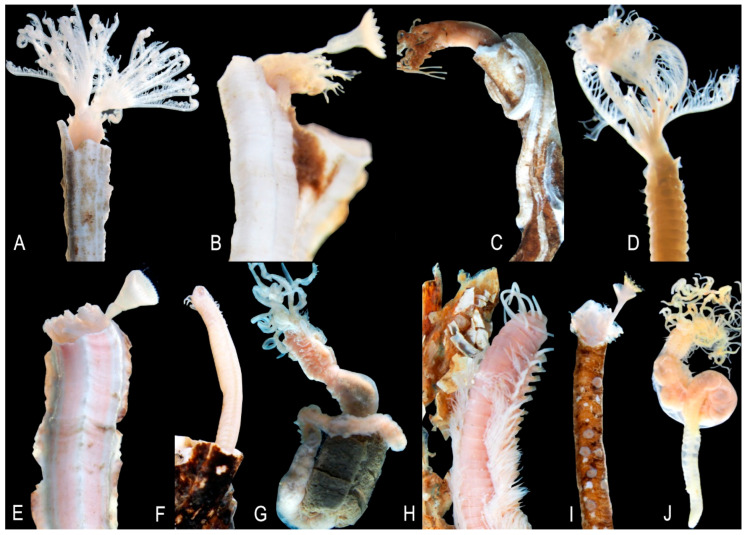
Some polychaete species are associated with the shells of *Atrina fragilis*. (**A**) *Protula tubularia*, (**B**) *Serpula concharum*, (**C**) *Terebella lapidaria*, (**D**) *Pseudopotamilla saxicava*, (**E**) *Serpula vermicularis*, (**F**) *Syllis hyalina*, (**G**) *Amphitrite rubra*, (**H**) *Eunice vittata*, (**I**) *Hydroides norvegica*, (**J**) *Polycirrus* sp.

The two dominant species on all *A. fragilis* shells were the serpulid polychaetes *Protula tubularia* and *Serpula concharum*, comprising nearly 30% of the total epifaunal populations (Figure 7). A total of 29 species had a dominance value lower than 1%. Depending on whether *A. fragilis* was alive or dead, changes in the dominant species on the shell were observed; *Vermiliopsis infundibulum, Terebella lapidaria*, and *Gnathia* sp. were the most dominant on dead shells. The species occurring most frequently on shells were *P. tubularia* (present on 75% of individuals), *T. lapidaria* (58%), *Pseudopotamilla saxicava* (58%), and *S. concharum* (58%) (Figure 7 and Figure 8). A total of 25 species were found on shells only once. Serpulids were found attached to the surface of shells, whereas terebellids, syllids, cirratulids, and sabellids were embedded in the layers of shells or occupied empty serpulid tubes. Polynoid, nereidid, and lumbrinereidid were found among serpulid tube aggregations.

**Figure 9:**
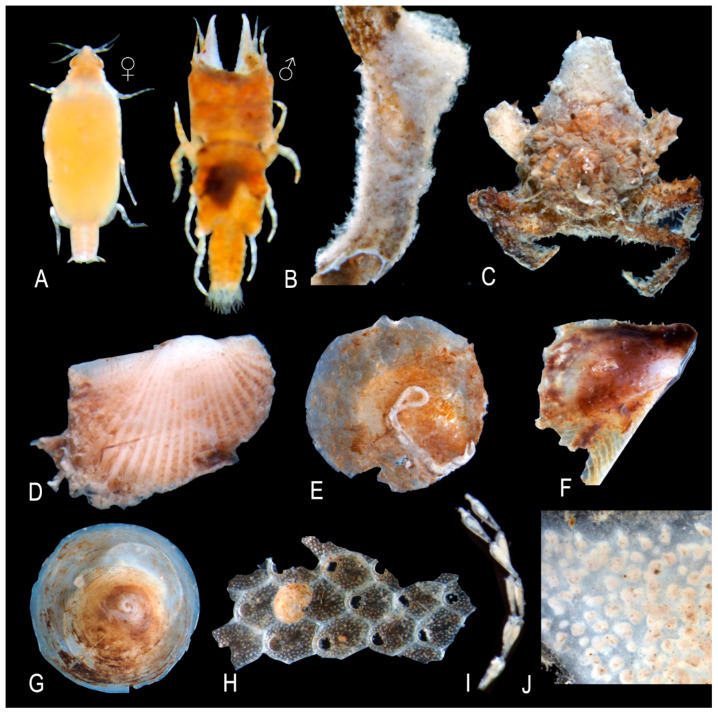
Some epifaunal species associated with the shells of *Atrina fragilis*. (**A**) Male and female individuals of *Gnathia* sp., (**B**) *Prosuberites longispinus*, (**C**) *Pisa nodipes*, (**D**) *Striarca lactea*, (**E**) *Anomia ephippium*, (**F**) *Pteria hirundo*, (**G**) *Calyptraea chinensis*, (**H**) *Arthropoma ceciliii*, (**I**) *Benedenipora* sp., (**J**) *Didemnum* sp.

The other most speciose taxonomic group found on the shells of *A. fragilis* was Mollusca; the majority of species belonged to the class Bivalvia and one species to Gastropoda. All were found attached to the shells. While the frequency and dominance values of the species were relatively low, the most abundant species was *Anomia ephippium*, and the least abundant species was *Calyptraea chinensis* (Figure 9). *Pteria hirundo* was found attached to the other species (ascidians and cnidarians) associated with *A. fragilis*. Species in other taxonomic groups were found attached to shell surfaces (sponge, bryozoans, and ascidians) or living among the tubes of polychaetes (crustaceans).

**Figure 10:**
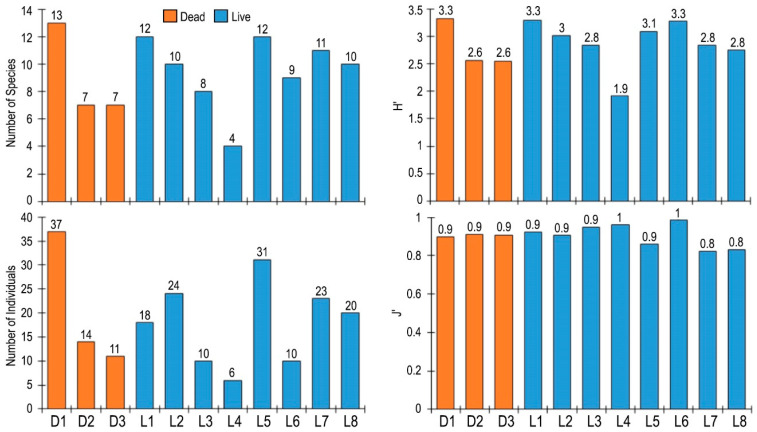
The number of species and individuals, and the diversity (H′) and evenness (J′) values of faunal communities associated with shells of dead (D) and live (L) individuals of *Atrina fragilis*.

The community parameters (number of species, number of individuals, diversity and evenness index) did not differ between the shells of dead and live individuals (Student *t*-test, *p* > 0.05). The average values of the community parameters were similar for dead and live individuals. For example, the average diversity index value was 2.81 (±0.26 SH) for dead individuals and 2.88 (±0.15 SH) for live individuals. The number of species varied from 4 to 13, the number of individuals from 6 to 37, and the diversity and evenness indices from 1.9 to 3.3 and 0.8 and 1, respectively (Figure 10).

**Figure 11:**
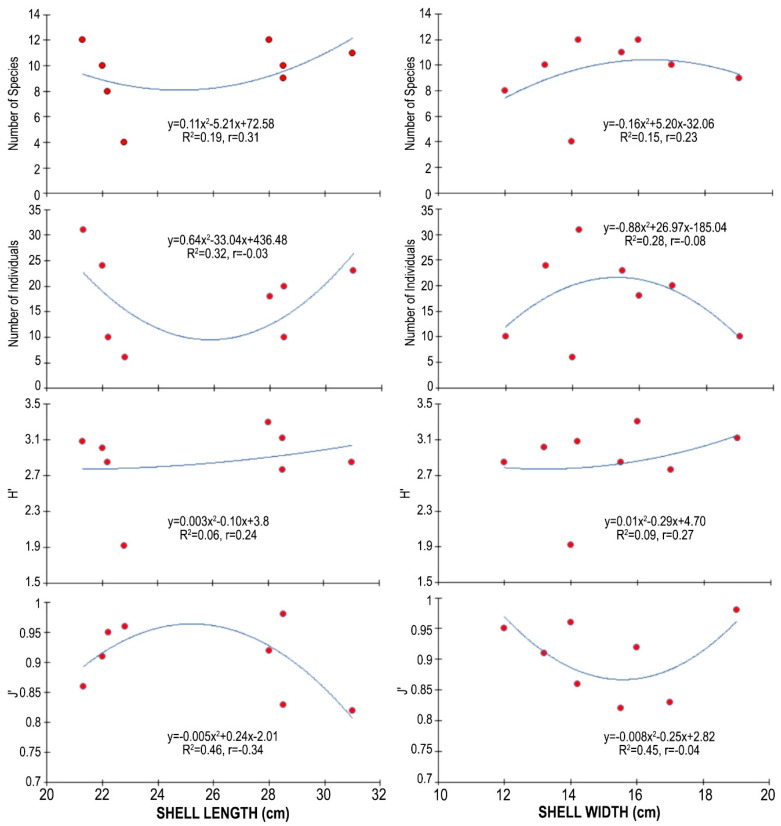
The relationships between shell length and width of *Atrina fragilis*, and the number of species, number of individuals, and the diversity (H′) and evenness (J′) values of the associated fauna.

Although the sample size is still too small to draw a reliable conclusion, the available data indicate a polynomial relationship between shell length and width, and the number of species and individuals of associated fauna. It appears that the number of species and individuals of associated fauna decreased as the shell length increased up to a certain point (24–26 cm), after which it gradually increased. The opposite was true for the shell width: the number of species and individuals of associated fauna increased up to a certain point (15–16 cm) and then decreased. The diversity index value increased with increasing shell length and width. However, the evenness index value decreased with increasing shell length, but increased with increasing shell width (Figure 11).

**Figure 12:**
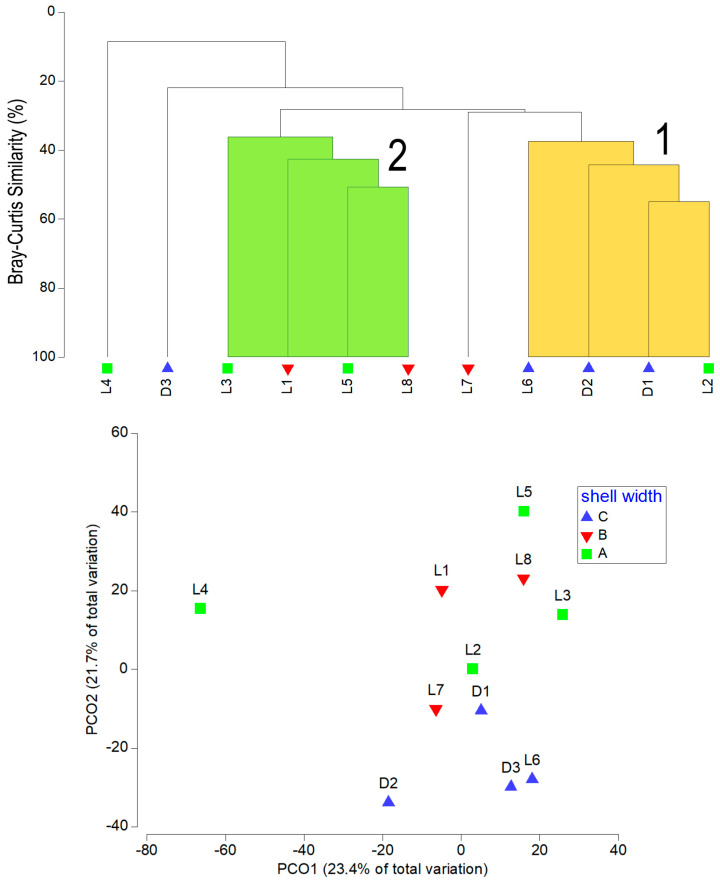
Dendrogram and principal coordinate plot (PCO) based on faunal abundance data associated with the shells of dead (D) and live (L) individuals of *Atrina fragilis*, which are categorized by width class (A = 12–15 cm, B = 15–18 cm, C = >18 cm).

**Figure 13:**
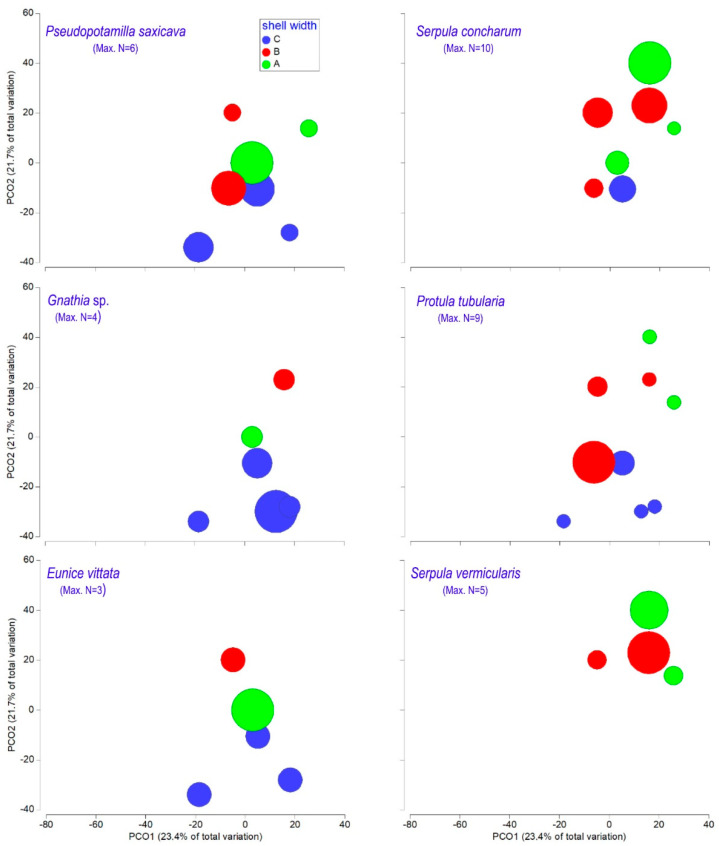
Biplot of the principal coordinate analysis with the abundances of some important species superimposed. The size of the circles is related to increasing abundance values.

The shell width appears to be an important factor in shaping species assemblages on shells (ANOSIM test: r = 0.74, *p* < 0.05). Two distinguishable groups (groups 1 and 2) can be seen on both the dendrogram and the PCO graph (Figure 12). The first two axes of the PCO graph explained 45.1% of the total variance. The first assemblage group (group 1, average similarity 43%) contained samples with shell widths greater than 18 cm (group C). The second group (group 2, average similarity 41%) contained two samples with shell widths between 12 and 15 cm (group A) and two samples with shell widths between 15 and 18 cm (group B). According to SIMPER, the species that contributed most to group 1 were *Pseudopotamilla saxicava* (24.4%), *Eunice vittata* and *Gnathia* sp. (both 16.4%), while those that contributed most to group 2 were *Serpula concharum* (31.1%), *S. vermicularis* (21%) and *Protula tubularia* (17.7%). The abundance levels of these species in the samples contributed significantly to the formation of the assemblages (Figure 13).

## 4. Discussion

Records of *Atrina fragilis* along the coasts of Türkiye are very rare and have only been mentioned in a few papers. The first record of this species (cited as *A. pectinata*) in Turkish waters was provided by Marion [36], who discovered a single living individual in the Istanbul Strait between Tophane and Kızkulesi, at 42 m depth. This represents the northernmost location at which this species has been found in the eastern Mediterranean. Demir [14] later reported the species in the Levant, Aegean, and Marmara Seas, without providing any locality data. He also stated that a variety of this species, A. *fragilis* var. ex form *spinulosa*, inhabits only the Aegean and Marmara Seas. However, Albayrak et al. [37] failed to find this species at any of the 254 stations they surveyed across the Sea of Marmara and questioned the presence of *A. fragilis* and some other species (e.g., *Pinna rudis*) in the region. Nevertheless, Çinar et al. [29] confirmed the presence of this species in the region, finding it to be relatively common at 40–50 m depths, at least around the southern Marmara Islands. It was indicated that stations with relatively dense beds of *A. fragilis* exhibited higher soft-bottom macrobenthic species diversity (over 55 species and a Shannon diversity index value of over 3.5), dominated by the ophiuroids *Amphiura filiformis* (O.F. Müller, 1776) and *Ophiura ophiura* (Linnaeus, 1758), the holothuroid *Paraleptopentacta elongata* (Düben & Koren, 1846), and the fish *Serranus hepatus* (Linnaeus, 1758), forming species-specific assemblages that differed from those in areas without *A. fragilis* [29]. This is consistent with previous findings indicating that dense *Atrina* beds can influence the physical and biological conditions of the environment, thus promoting faunal biomass and diversity locally in soft substrata [12,20,38]. The species composition and mean abundance of common soft-bottom taxa were proved to be different inside and outside of *Atrina* patches in New Zealand [12]. Hewitt et al. [39] suggested that soft-bottom macrofaunal assemblages were related to the spatial arrangement of *A. zelandica* (J. E. Gray, 1835) rather than to its density alone.

The present study indicated that both dead and living individuals of *A. fragilis* were only found within a specific depth range in the Sea of Marmara, from 42 to 67 m. Calculations based on living individuals showed that the *A. fragilis* population density varied from 31 ind. km^−2^ (station 6) to 469 (station 4) ind. km^−2^ in the region. Stations 4 and 5, which are located off the southern part of Paşa Limanı at depths 41–45 m, appear to have a relatively extensive *A. fragilis* bed. Both juvenile and adult individuals of this species were reported in the northern Adriatic between 1982 and 1994, and the density of the former was estimated maximally as 19,284 ind.km^−2^ and that of the latter as 5489 ind.km^−2^ [13]. In the same sea, the maximum density of *A. fragilis* was estimated as 250 ind.km^−2^ before dredging and 40 ind.km^−2^ after dredging [17]. Howson et al. [38] estimated its density to be between 2 ind.m^−2^ and 4 ind.m^−2^ in the densest patches around Canna Island, which is located in the northern part of the UK. However, Solandt [40] indicated the scarcity of its population in the region.

Scallop dredging and trawling have been implicated in the decline of *Atrina fragilis* and other pinnid populations [13,16,17,40,41]. These destructive fishing methods have been shown to reduce the abundance of *A. fragilis*, as well as the sponge *Geodia cydonium* (Linnaeus, 1767), the mollusks *Pecten jacobaeus* (Linnaeus, 1758), and *Neopycnodonte cochlear* (Poli, 1795), and sea-cucumbers in the Adriatic Sea [42]. In the same region, the density of *A. fragilis* changed considerably in trawled areas (0.03 ind.km^−1^) and untrawled areas (5.5 ind.km^−1^) [13]. Rapido trawling for scallops in the Gulf of Venice has resulted in a significant reduction (87%) in the *A. fragilis* abundance [17]. A dense aggregation of dead shells (2326 ind.km^−2^) was noted in various parts of the Adriatic Sea, indicating the effects of dredging and trawling on its survival [13]. While this has not yet been reported for *A. fragilis*, some *Atrina* species, such as *A. pectinata* (Linnaeus, 1767), have experienced mass mortality (60–90%) due to viral diseases in Japan during the late spring and summer of 2003 and 2004 [43].

Due to its fragile shell, *A. fragilis* is susceptible to any form of physical disturbance, such as trawling [40,44]. Bottom-trawling is prohibited in the Sea of Marmara, including the Istanbul and Çanakkale Straits, by national legislation. However, another destructive fishing device, the beam trawl (algarna), is widely used for shrimp fishing at depths greater than 50 m, and the empty shells found in the present study may indicate the impact of this activity. The composition of discards from this type of fishing activity in the region has not been studied in detail; however, Zengin and Akyol [45] reported an individual of the family Pinnidae (presumably *A. fragilis*) from algarna hauls in the southern Marmara Sea at depths between 40 and 80 m. The impact of this fishing activity on *A. fragilis* populations in the region is largely unknown, but it is highly likely that extensive *A. fragilis* beds in the Sea of Marmara might have been completely destroyed before they could be discovered. It currently has no protected status in either the Mediterranean Sea or Türkiye, whereas it is strictly protected in the United Kingdom [16,40]. It is currently considered one of the most endangered mollusks in the UK [40]. Given its ecological role and vulnerability, we argue that it should be protected in all areas in which it lives. Studies have shown that the population density and shell sizes are much higher for this species in protected areas than in areas subject to trawling [16].

The shell lengths of live *A. fragilis* individuals in the Sea of Marmara ranged from 21.3 to 31 cm (mean: 25.31 cm). Much smaller (14.17 cm) and larger (46.53) sizes (mean: 34.86) were encountered in the Thermaikos Gulf in the Aegean Sea [16]. The maximum shell width measured was 19 cm in the Sea of Marmara and 28.7 cm in the Aegean Sea [16]. The maximum recorded shell length for this species is 48 cm in UK waters [38].

The associated fauna of *A. fragilis* shells has not yet been studied in detail. The only available information was provided by Hall-Spencer et al. [17], who emphasized the importance of the shells for scallop recruitment in the Adriatic Sea as they provide a substratum for scallops to attach to. They also stated that shell surfaces provide suitable substrata for the settlement of many invertebrates, including juvenile pectinids, serpulids, hydroids, and bryozoans. Šimunović [13] reported the presence of *Anomia ephippium, Calyptraea chinensis, Crepidula unguiformis* (Lamarck, 1822), *Capulus ungaricus* (Linnaeus, 1758), *Mimachlamys varia* (Linnaeus, 1758), and *Aequipecten opercularis* (Linnaeus, 1758) on *A. fragilis* shells in the Adriatic Sea. However, the shells of other pinnid species, such as *Pinna nobilis*, are known to host diverse epifaunal communities. Rabaoui et al. [1] recorded 146 species on *P. nobilis* along the Tunisian coast. Çinar et al. [3] found ten sessile and four motile species on *P. nobilis* in the Sea of Marmara. The present study, which involved a limited number of *A. fragilis* individuals, encountered 47 invertebrates on the shells; the most dominant species were *Protula tubularia* and *Serpula concharum*. Epifaunal diversity and abundance changed according to the shell size of *A. fragilis*, in accordance with the data provided by Banach Esteve et al. [46] and Çinar et al. [3].

## 5. Conclusions

This study provides a comprehensive overview of the current population structure, spatial distribution, and shell-associated macrofaunal communities of *Atrina fragilis* in the Sea of Marmara. Our findings suggest that the species exhibits a patchy and fragmented distribution in the region, primarily due to habitat and depth specificity. The distribution of this species in the region remains largely unknown. This study is the first to analyze the population characteristics of this species along the coasts of Türkiye. *Atrina fragilis* shells support a diverse assemblage of macrofaunal organisms, highlighting their importance as biogenic structures that contribute to local biodiversity.

Given the ecological significance of *A. fragilis* as both a habitat-forming and sensitive benthic species, conservation measures should prioritize the protection of its habitats and the regulation of activities that lead to seabed disturbance. The presence of dead shells in the present study suggests that the species may have been subject to physical disturbance in the Sea of Marmara. The relatively low population density at the study sites underscores the species’ vulnerable conservation status and the pressing need for habitat protection and further monitoring. Therefore, any deterioration in their populations could have cascading effects on the associated benthic community. Future research should focus on long-term monitoring programs and the potential impact of climate change on the persistence of *A. fragilis* populations.

## Figures and Tables

**Table 1:** The start and end coordinates and depths (m) of the stations where *Atrina fragilis* was found, along with the sampling date.

Stations		Coordinates	Depth	Date
1	Start	40°33.97′ N–27°44.52′ E	67	3 April 2021
	End	40°34.43′ N–27°46.15′ E	66	
3	Start	40°26.17′ N–27°37.40′ E	43	5 April 2021
	End	40°25.93′ N–27°35.84′ E	45	
4	Start	40°26.19′ N–27°37.31′ E	42	10 September 2021
	End	40°25.99′ N–27°35.75′ E	45	
5	Start	40°27.24′ N–27°34.59′ E	43	13 September 2021
	End	40°26.53′ N–27°33.16′ E	45	
6	Start	40°31.05′ N–27°38.67′ E	52	12 December 2021
	End	40°31.11′ N–27°40.21′ E	54	

**Table 2:** List of the species associated with the shells of dead (D) and live (L) *Atrina fragilis* individuals at stations 4 and 5. The fauna associated with the shells of three dead and nine live *A. fragilis* individuals were treated separately at station 4, while all faunal data on the shells of six dead and one live *A. fragilis* individuals were grouped together at station 5.

Stations	4												5
**Dead (D**)**/Live (L**) **Individual**	**D**	**D**	**D**	**L**	**L**	**L**	**L**	**L**	**L**	**L**	**L**	**L**	**6D, 1L**
**PORIFERA**													
**Agelasida**													
*Prosuberites longispinus* Topsent, 1893	1	-	-	-	-	-	-	-	1	1	1	-	-
**CNIDARIA**													
**Hydrozoa**													
*Clytia paulensis* (Vanhöffen, 1910)	-	-	1	-	-	-	-	-	-	-	-	-	-
**Anthozoa**													
*Alcyonium palmatum* Pallas, 1766	-	-	-	-	-	1	-	-	-	-	-	-	-
*Epizoanthus arenaceus* (Delle Chiaje, 1841)	1	-	-	-	-	-	-	-	-	-	-	-	-
**POLYCHAETA**													
**Polynoidae**													
*Harmothoe* sp.	-	-	-	-	-	-	-	-	-	-	-	-	1
**Euphrosinidae**													
*Euphrosine foliosa* A. and M. Edwards, 1833	-	-	-	-	-	-	-	-	-	-	-	-	1
**Syllidae**													
*Myrianida* sp.1	-	-	-	-	-	-	-	-	1	-	-	-	1
*Myrianida* sp.2	1	-	-	-	-	-	-	-	-	-	-	-	-
*Syllis hyalina* Grube, 1863	6	-	-	2	1	-	-	-	-	-	1	-	2
**Nereididae**													
*Composetia hircinicola* (Eisig, 1869)	1	-	-	-	-	-	-	-	-	-	-	-	-
**Lumbrineridae**													
*Abyssoninoe hibernica* (McIntosh, 1903)	-	-	-	-	-	-	-	-	1	-	-	-	-
**Eunicidae**													
*Eunice vittata* (Delle Chiaje, 1828)	1	1	-	1	3	-	-	-	1	-	-	-	3
**Spionidae**													
*Polydora* sp.	-	-	-	-	-	-	-	-	-	1	-	-	-
**Cirratulidae**													
*Aphelochaeta* sp.	-	-	-	-	-	-	-	-	-	1	-	-	-
*Cirratulus* sp.	-	-	-	-	-	-	-	-	-	-	-	-	1
*Cirriformia* sp.	-	-	-	-	1	-	-	-	-	-	-	-	-
*Monticellina* sp.	-	-	-	-	1	-	-	-	-	-	-	-	-
**Maldanidae**													
*Metasychis gotoi* (Izuka, 1902)	-	-	-	-	-	-	-	-	-	-	-	-	1
**Terebellidae**													
*Amphitrite rubra* (Risso, 1828)	-	-	-	-	-	-	2	-	-	1	1	-	7
*Terebella lapidaria* Linnaeus 1767	7	-	1	-	4	1	-	4	2	-	1	-	3
*Neoamphitrite figulus* (Dalyell, 1853)	-	-	-	-	-	-	-	1	-	-	-	-	-
*Polycirrus* sp.	-	-	1	-	-	-	-	-	-	-	-	-	-
**Sabellidae**													
*Branchiomma bombyx* (Dalyell, 1853)	-	-	-	-	3	-	-	1	-	-	-	-	1
*Pseudopotamilla saxicava* (Quatrefages, 1866)	4	3	-	1	6	1	-	-	1	4	-	-	-
**Serpulidae**													
*Hydroides norvegica* Gunnerus, 1768	-	-	-	1	-	-	-	-	-	-	-	-	4
*Protula tubularia* (Montagu, 1803)	3	1	1	2		1	-	1	1	9	1	-	21
*Serpula concharum* Langerhans, 1880	4	-	-	5	3	1	-	10	-	2	7	-	1
*Serpula vermicularis* Linnaeus, 1767	-	-	-	1	-	1	-	4	-	-	5	-	2
*Vermiliopsis infundibulum* (Philippi, 1844)	5	4	-	-	-	-	-	-	-	-	-	-	-
**CRUSTACEA**													
**Isopoda**													
*Gnathia* sp.	2	1	4	-	1	-	-	-	1	-	1	-	1
**Decapoda**													
*Pisa nodipes* Leach, 1815	-	-	-	-	-	1	-	-	-	-	-	-	-
**MOLLUSCA**													
**Gastropoda**													
*Calyptraea chinensis* (Linnaeus, 1758)	-	-	-	-	-	-	-	-	-	-	1	-	-
**Bivalvia**													
*Anomia ephippium* Linnaeus, 1758	-	1	2	-	-	-	-	-	-	1	-	-	2
*Musculus subpictus* (Cantraine, 1835)	-	-	-	-	-	-	-	2	-	-	-	-	-
*Ostrea edulis* Linnaeus, 1758	-	-	-	-	-	3	-	2	-	-	-	-	-
*Pteria hirundo* (Linnaeus, 1758)	-	-	-	-	-	-	-	3	-	-	-	-	-
*Striarca lactea* (Linnaeus, 1758)	-	-	-	1	-	-	-	-	-	-	-	-	1
**BRYOZOA**													
**Cheliostomatida**													
*Arthropoma cecilii* (Audouin, 1826)	-	-	-	-	-	-	-	-	-	1	-	-	-
*Callopora dumerilii* (Audouin, 1826)	-	-	-	-	-	-	-	-	-	1	-	-	-
*Smittoidea reticulata* (MacGillivray, 1842)	-	-	-	-	-	-	-	1	-	-	-	-	-
**Ctenostomatida**													
*Benedenipora* sp.	-	-	-	1	-	-	1	-	-	-	-	-	-
**ECHINODERMATA**													
**Ophiuroidea**													
*Amphiura chiajei* Forbes, 1843	-	-	-	-	-	-	-	-	-	1	-	-	-
**TUNICATA**													
**Ascidiacea**													
Tunicata sp.1	1	3	-	1	1	-	2	1	-	-	-	-	1
*Didemnum* sp.	-	-	-	1	-	-	-	1	-	-	1	1	1
*Ascidiella aspersa* (Müller, 1776)	-	-	-	-	-	-	1	-	-	-	-	-	1
*Ascidia virginea* Müller, 1776	-	-	1	1	-	-	-	-	-	-	-	-	-
*Phallusia mammillata* (Cuvier, 1815)	-	-	-	-	-	-	-	-	1	-	-	-	-

## Data Availability

The authors confirm that the data supporting the findings of this study are available within the article.
